# ERP Indicators of Self-Pain and Other Pain Reductions due to Placebo Analgesia Responding: The Moderating Role of the Fight-Flight-Freeze System

**DOI:** 10.3390/brainsci11091192

**Published:** 2021-09-10

**Authors:** Arianna Vecchio, Vilfredo De Pascalis

**Affiliations:** Department of Psychology, La Sapienza University of Rome, 00185 Rome, Italy; vilfredo.depascalis@uniroma1.it

**Keywords:** phasic pain, empathy pain, Reinforcement Sensitivity Theory, FFFS, active avoidance, EEG, N1, P2, P3

## Abstract

This study evaluates the modulation of phasic pain and empathy for pain induced by placebo analgesia during pain and empathy for pain tasks. Because pain can be conceptualized as a dangerous stimulus that generates avoidance, we evaluated how approach and avoidance personality traits modulate pain and empathy for pain responses. We induced placebo analgesia to test whether this also reduces self-pain and other pain. Amplitude measures of the N1, P2, and P3 ERPs components, elicited by electric stimulations, were obtained during a painful control, as well as during a placebo treatment expected to induce placebo analgesia. The placebo treatment produced a reduction in pain and unpleasantness perceived, whereas we observed a decrease in the empathy unpleasantness alone during the empathy pain condition. The moderator effects of the fight-flight-freeze system (FFFS) in the relationships linking P2 and P3 amplitude changes with pain reduction were both significant among low to moderate FFFS values. These observations are consistent with the idea that lower FFFS (active avoidance) scores can predict placebo-induced pain reduction. Finally, in line with the revised Reinforcement Sensitivity Theory (r-RST), we can assume that phasic pain is an aversive stimulus activating the active-avoidance behavior to bring the system back to homeostasis.

## 1. Introduction

Pain is a complex psychophysical phenomenon characterized by unpleasant sensory and emotional experience in which the sensorial-discriminative component of a complex system of nerve circuits [1], defined as the pain matrix [2,3,4], is occasionally not necessary to generate this complex phenomenon [5]. Because pain is a subjective and individual experience, it is sometimes difficult to discriminate the unpleasant component of the noxious stimulus from a painful one. In this regard, Fields [6] has defined pain as the result of two significant components, algosity, and sensory unpleasantness, transducing the noxious stimulation in input by the sensory-discriminative pathway. However, researchers have also shown that the motivational and affective properties of a painful experience are part of the pain phenomenon [6,7,8,9,10]. Additionally, the social dimension related to the sharing of painful experiences has been suggested as an essential factor influencing the pain experience [11] beyond the individual elaboration of the organic damage suffered [12].

In terms of approach and avoidance motivational personality traits, pain can be conceptualized as a powerful, dangerous stimulus that generates behavioral avoidance. Moreover, research has suggested that motivational personality traits [13] are the psychological factors that play an essential role in pain reduction induced by placebo treatment [14]. Personality traits as a positive approach (positive orientation, resilience, and extroversion) can explain the complexity of the placebo antalgic response [15], which is known to be associated with the release of endogenous opioids [16] and dopamine in the reward circuits (basal ganglia), and indirectly acts on the prefrontal cortex [17]. One of the most famous neuroscience theories of personality is the Revised Reinforcement Sensitivity Theory (r-RST) [18,19,20,21]. This theory has conceptualized as the behavioral approach system (BAS), the behavioral inhibition system (BIS), and the fight-flight-freeze system (FFFS) as biology-based traits regulating approach-avoidance behavior. Corr and Cooper [22] have recently developed and validated the Reinforcement Sensitivity Theory-Personality Questionnaire (RTS-PQ) in this framework. This questionnaire measures six factors, two unitary defensive factors, the FFFS (related to fear), BIS (related to anxiety), and four BAS factors. The BAS is a multidimensional system [18] composed of the following facets. Reward interest (RI) and (GDP) sub-components are associated with the motivation to obtain a reward (wanting behavior). Reward reactivity (RR) and impulsivity (Imp) are associated with the hedonic experience of sensorial reward (liking sensation) that is obtained with the appropriation of the rewarding object. The wanting is related to dopaminergic neurotransmission in the ventral striatum [23]. At the same time, the liking component is associated with the hedonic hotspots for opiates present in the exit to the pale ventral globe [24,25].

The BIS is associated with anxiety, the inhibition of ongoing behavior, and scanning of the environment. In situations that cause anxiety and fear, the hippocampus amplifies the neural representation of adverse events to adopt a more adaptive behavior in the worst case possible and to preserve the organism [19] better. Furthermore, in the rRST, the original BIS is developed into a fight-flight-freeze system (FFFS) related to responses to aversive stimuli. The FFFS is the primary system responsible for fear responses and is activated only in the case of active avoidance of a threatening stimulus. This system mediates fear reactions to conditioned and unconditioned aversive stimuli, and it is responsible for active avoidance and escape behaviours [19,21]. If the situation requires an attack on the threat, both the BIS and FFFS are activated (fight). If the scary object is sufficiently distant, the freezing conduct (freezing block) or active avoidance behavior (flight) is elicited to avoid attracting the attention of the predator [19]. Finally, in the revised RST, the function of the BIS is primarily to detect and resolve conflicts, as it may be a case concerning a dangerous situation in which both the BAS and FFFS are activated. The FFFS facilitates the response to the fear-eliciting stimuli, while BAS can contrast this response.

In line with this framework, placebo-induced pain relief can be seen as a rewarding process related to BAS [26,27], which is an opponent process that intervenes to interrupt a dangerous stimulus [28] that generates conflict [29] to bring the system back to homeostasis [30]. Furthermore, in terms of BIS parallel measures, and according to Sternbach [31], anxiety significantly influences the pain experience because higher levels of stress-anxiety are related to higher levels of pain intensity and are inversely associated with the placebo analgesic effect [32]. Additionally, it is essential to consider how social interaction and empathy traits become relevant during a therapeutic relationship. In fact, regarding prosocial behavior, empathy allows us to share and understand the emotions of others [33,34]. In this sense, it could be a significant motivational factor to help pain-experiencing individuals reach pain relief. For example, when an individual sees another person (a simulator) reporting relative placebo pain relief following the same painful treatment, it can also induce a placebo analgesic effect in the observer [35]. Besides pain, social interaction, or in this case, empathy experience, can be modulated by the intake of analgesics. For example, in a social interaction contest, when an individual observes another suffering, ingestion of painkillers (e.g., paracetamol) decreases empathy for the pain response [36]. Therefore, it can be supposed that the analgesic effect induced by a placebo can reduce empathy for pain as a drug.

### Electrophysiology of Pain and Empathy for Pain Induced by Placebo: Aims of the Study

One of the aims of this study was to evaluate the association of approach/avoidance personality traits [22] with both phasic pain and empathy for pain changes induced by a placebo analgesia treatment [37,38]. Another aim was to determine how FFFS (fear, active avoidance) and BIS (passive avoidance) can affect placebo analgesia changes in phasic pain’s sensory and affective components and empathy for pain.

Neuropsychological research has shown that phasic pain stimulation elicits event-related potential (ERP) responses, in which N1 and P2 components are associated with the sensory processes [39,40,41], while later P3 [42] and P4 [43] components are associated with cognitive functions [44]. The N1 wave (120–160 ms) reflects the first phase of sensory processing as stimulus attention and discrimination of the nociceptive stimulus [45]. The P2 wave (200–300 ms) relates to a later stage of the sensory elaboration of the nociceptive input [46]. In addition, the N160 wave originating in the parietal operculum [47], has been found to modulate the placebo effect during nociceptive perception [48]. Later, Rütgen and colleagues [38] studied placebo-induced modulation of pain and empathy for pain. They observed an amplitude reduction of the P2 component at the central scalp lead (Cz) in both conditions. This site is located near the medial cingulate cortex (MCC) and is sensitive to both the modulation of perceived pain [49] and empathic pain responses [50]. The MCC is the site of numerous μ-opioid receptors that modulate pain perception [51], and for this reason, the P2 component reaches its maximum amplitude on the Cz site [52]. Research has shown that the modulation of the P2 and P3 waves reaches the maximum amplitude at the Cz site and amplitude changes in these two components are valid indicators of placebo-induced phasic pain [47,53] and empathy pain reductions [38,54]. Moreover, these studies have suggested that the ERPs’ P2, N2, and P3 components reflect the activation of Aδ fibers that generate a consecutive activation of C fibers associated with the engenders of an ultra-late P4 wave (400–600 ms) [55,56]. In this vein, empathy-related studies [43,57,58,59] have suggested that the P3 component is associated with empathy pain processing. Specifically, research has highlighted a more significant P450 response to empathic pain than neutral stimuli on the left central-parietal site (CP3) near the motor cortex area [43,57,59]. Similar results were reported in experiments that combined phasic self-pain and empathy for pain [60]. Subsequently, González-Franco [61] reproduced the findings reported by Meng and colleagues [57], Li and Han [58], and Fan and Han [59] in a virtual reality simulation, in which participants experienced a virtual threat on their backhand. These authors observed a higher P450 amplitude on the CP3 site, suggesting that this response may have the same function of readiness potential, such as a natural response emitted by an individual when a forthcoming stab threatens their hand.

According to Gallese and Sinigaglia [62], empathy is a simulation process based on the projection of one’s own subjective experience concerning the emotion of other individuals [62,63]. In this regard, we are interested in deepening the study of the ERP processing associated with phasic pain and empathy for pain induced by placebo analgesia. We hypothesized that placebo treatment, compared to a painful control, can, like a drug, reduce both phasic pain and empathy for pain sensations, and this effect should be parallel to decreasing amplitudes of the N1, P2, N2, P3, and P4 components of the ERPs.

## 2. Materials and Methods

### 2.1. Participants

In this study, 63 right-handed university student volunteers (32 women: mean [M] = 21.56, standard deviation [SD] = 2.41, range 18–27 years; men: M = 23.03, SD = 2.63, range 19–29 years) were included. Handedness was measured using the Italian version of the Edinburgh Handedness Inventory [64,65]. The participants did not report a history of drugs (psychotropic substances, corticosteroids, or painkillers), illnesses, neurological, psychopathological, psychiatric problems, or color-blindness. Furthermore, the female participants on their menstrual periods were scheduled for electroencephalogram (EEG) recordings between the 5th and 11th day after the onset of menses to avoid possible impairment on the EEG recording [66]. All participants were asked to refrain from caffeine and smoking two hours before the EEG study sessions [67]. Participants were subsequently invited individually to the laboratory and informed of the nature of the study.

This study was approved by the Institutional Review Board (IRB) of the Department of Psychology, “La Sapienza” University of Rome, and was conducted according to the Declaration of Helsinki (1964).

### 2.2. Questionnaire

We administered the RST-PQ [22] to measure the personality traits of interest. The RST-PQ provided measures of the three significant approach/avoidance motivational dispositions: the BIS (Cronbach’s α value = 0.88), FFFS (Cronbach’s α value = 0.79), and four facets of the BAS. The four BAS facets are (1) BAS-GDP (Cronbach’s α value = 0.85), related to the individual’s emotional/motivational activation to pursue a planned behavior for achieving a goal. (2) BAS-RI (Cronbach’s α value = 0.79), related to the anticipation and planning processes to reach the reward. (3) BAS-RR (Cronbach’s α value = 0.78), related to the hedonic component of the reward after its consumption. (4) BAS-I (Cronbach’s α value = 0.77), associated with reward sensitivity. Furthermore, we also calculated the sum of the BAS-GDP, BAS-RI, BAS-RR, and BAS-I scores to measure the total BAS and labeled it as BAS-TOT (Cronbach’s α = 0.87).

Participants filled the State-Anxiety Inventory (STAI-Y1) [68] after each experimental task. Finally, we also issued a set of other questionnaires not considered in the present study.

### 2.3. Experimental Task and Trial Structure

**Figure 1:**
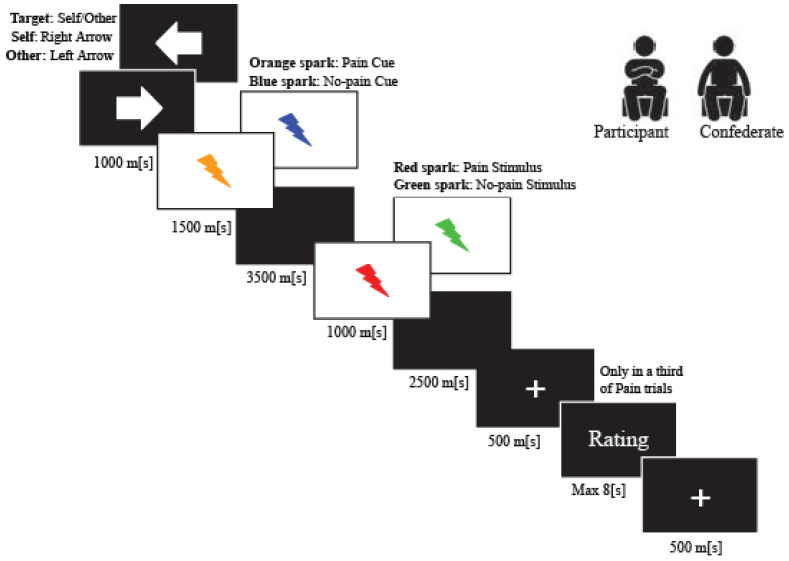
Trial Structure.

In this experiment, to study the modulation of phasic pain induced by placebo analgesia, each participant was exposed to individually calibrated short-painful electrical stimulations. Furthermore, to assess the modulation of the empathetic pain response induced by placebo analgesia, we used the empathy for pain paradigm [37,38], wherein, based on the evaluation of perceived pain, the participant was asked to infer the pain perceived by another participant (the confederate) seated nearby. Each participant was exposed to short, non-painful electrical stimulations randomized with painful stimuli to control the painful habituation. The empathy for pain paradigm was executed by e-prime 2.0 software. The intensity of electrical pain and non-painful stimulation never exceeded an intensity value of 10 mA and was delivered by a Digitimer DS5 Isolated Bipolar Constant Current Stimulator (Digitimer Clinical and Biomedical Research Instruments). To study the response of the nociceptive component of only the phasic, or in this caseelectrical pain, a concentric wasp point electrode with a 7 mm diameter and a central platinum pin (WASP electrode, Specialty Developments) positioned at the back of the participant’s right hand was used. Research has shown that this electrode typology can selectively stimulate the A-δ fibers and A-C terminals [69,70]. Therefore, this type of electrode makes it possible to detect the sensory evoked potentials of phasic pain. The experimental task was composed of 36 trials randomly presented for each condition: painful and non-painful stimulation to the participant and painful and non-painful stimulation to the confederate (Figure 1).

Initially, we presented an arrow (1500 ms) indicating the target of the upcoming electric stimulus (arrow to the right: stimuli delivered to the participant; arrow to the left: stimuli delivered to the confederate). We then presented an anticipatory cue (500 ms) indicating the intensity of the upcoming electric stimulus (orange sparkle for pain stimulus and blue sparkle for no-pain stimulation). After the anticipatory cue, we delivered the electrical impulse at the onset of the second visual stimulus (red spark for pain stimulus and green sparkle for non-pain stimulation), lasting 1000 ms.

In one-third of the painful trials, the participant expressed two values to measure experienced pain and unpleasantness, following the noxious stimulation (“How painful was this stimulus for you?”; “How unpleasant was this stimulus for you?”). Similarly, participants expressed two values to assess the empathy pain and empathy unpleasantness measures. In the latter case, the participants had to infer the values for pain and the unpleasantness felt by the confederate when she received the noxious stimulation (“How painful did it feel when the other person was stimulated?”; “How unpleasant did it feel when the other person was stimulated”). All measures were collected on a seven-point Likert scale.

### 2.4. Procedure

The study consisted of two experimental days. On the first testing day, the participant signed the informed consent and completed the RST-PQ [22]. On the second testing day, we introduced the participant and the confederate to each other for presentation in our laboratory. The confederate was always a woman, as well as the experimenter. Furthermore, it is important to specify that the confederate was not known to any of the participants.

Before administering the experimental task, all participants underwent a psychophysical pain calibration procedure [71] of electric pulse stimulation to determine a reliable electrical stimulation intensity for painful and non-painful stimuli [38]. After each impulse, the participant expressed a value on a seven-point Likert scale, ranging from 1 (“perceptible but no painful sensation”) to 7 (“worst pain ever perceived”). The painful stimuli were chosen as the stimulus rated by participants before the seven values as “6”, corresponding to significant but not unbearable pain. In contrast, the non-painful stimulus was chosen as the second value rated as “2” and was thus defined by the participant as a clearly perceptible stimulation, but not painful.

The behavioral test consisted of an experimental design balanced within groups for two treatments: placebo and pain (placebo), and only pain (control). Furthermore, we assigned participants to the treatments in a counterbalanced order across participants (placebo-control; control-placebo). In the placebo experimental treatment, we followed a double-blind procedure. The experimenter to each participant administered and presented, through a verbal suggestion, a capsule containing an inert substance as a powerful painkiller with a high or low analgesic dosage [72]. We employed this experimental protocol to avoid the analgesic capsule being detected as a sham treatment, given that the participants were all students in psychology courses. In this way, we tried to prevent any violation of expectations generated by uncertainty on the electrocortical responses. To increase the analgesic effect induced by the placebo, the experimenter tested the expectancy of the medication by asking the participant to introduce a value on a seven-point Likert scale asking the question: “How much do you expect this medication to be effective in reducing your pain?” We applied a pain manipulation procedure 20 min after pill administration to test the effectiveness of the placebo effect; during the pain manipulation, the experimenter delivered four stimuli evaluated by the participant during the calibration phase as a medium level of pain (3 or 4). However, we led participants to believe that the experimenter delivered stimuli that they had previously rated as extremely painful (i.e., a 6 rating score). We used this method to amplify the analgesic effect induced by the placebo [73]. After this conditioning procedure, we asked the participant to rate the treatment efficacy on a seven-point Likert scale, referring to the question: “How effective was this medication in reducing your pain?” Later, the experimenter informed the participants that the “analgesic capsule” would be effective for 50–60 min.

The experimenter informed all participants that the confederate would not receive any medication. This manipulation was necessary to evaluate how the analgesia induced by the placebo can modulate the empathy for pain response of the participant. Furthermore, according to Coll and colleagues [74,75], empathizing with a person means inferring a state of mind that is experienced first-hand. The participant and confederate were seated in the EEG-recording chamber. We asked them to avoid the direct observation of each other to elude the consequent emotion contagion induced by facial mimics, which could impair an empathic response. It was essential for the participant to infer the empathic response of the confederate based first-hand on the experienced painful feeling.

The experimental task lasted approximately 45 min, while the experiment in total lasted 1.9 h. We delivered painful and non-painful stimuli in a random order to both the participant and the confederate. We gave the state anxiety inventory [68] at the end of each experimental task to assess the influence of the state of anxiety.

The control treatment was the same as the placebo, but the experimenter did not administer the pill (placebo) to the participants. Finally, we debriefed the participants at the end of the two experimental conditions (placebo, control).

### 2.5. Electrophysiological Recordings

We recorded the behavioral and electrophysiological measures in a light-attenuated soundproof and electrically shielded room. Participants performed their behavioral task seated in a comfortable chair at an approximate 80 cm distance (visual angle, horizontal of 5.2° and vertical of 6.9°) to the 19″ LCD monitor (refresh 75 Hz, resolution 1400 × 900, 22.5 Cd/m^2^).

We recorded the EEG using a 40-channel NuAmps DC amplifier system (Compumedics Neuroscan Inc, Charlotte, NC, USA) set in a DC mode, with a gain of 200 (100 for eye channels) and a bandpass of 0.01–100 Hz (Butterworth zero-phase filter with 24 dB/octave roll-off). The data were recorded according to the standard international 10–20 system [76] at 30 scalp sites (Fp1, Fp2, F7, F8, F3, F4, FT7, FT8, T3, T4, FC3, FC4, C3, C4, CP3, CP4, TP7, TP8, T5, T6, P3, P4, O1, O2, Fz, FCz, Cz, CPz, Pz, Oz) using a32-tin electrodes stretch Lycra cap (Electro-Caps, Eaton, OH, USA). Each signal was filtered online using a 50 Hz notch filter and stored on a Neuroscan Acquire 4.3 system. We set the electrode impedance at less than 5 kΩ. The EEG signals were referenced to the earlobes using a digitally connected (A1 + A2)/2 (Neuroscan Acquire setting) pure tin electrode couple within a frequency range from 0–250 Hz and digitally stored with a sampling rate of 1000 Hz. We set the ground reference between the Fz and FPz midline sites. We placed additional electrodes to record vertical and horizontal electrooculograms (EOG). For the horizontal-EOG recording, we used a pair of tin electrodes placed 1 cm lateral to the outer canthus of each eye. For the vertical-EOG recording, we used a separate bipolar montage with electrodes set above and below the center of the left eye.

### 2.6. ERP Analysis

After the data acquisition phase, we analyzed the EEG signals offline using the Brain Vision Analyser software 2.1.0 (Brain Product GmbH, Gilching, Germany). We visually inspected the EEG signals in the pre-processing phase and rejected each portion of the EEG data showing ocular, muscular, or technical artifacts. At the same time, we interpolated the disconnected or noisy channel(s) using the triangular interpolation technique tool implemented in the Brain Vision Analyser Software 2.1.0. We used the method of Gratton and colleagues [77] for ocular corrections, while we removed the residual artifacts exceeding ±75 µV. To attenuate the signal noise, we filtered the EEG using a bandpass filter that excluded the frequency bands below the cut-off frequency of 0.1 Hz and higher than the cut-off frequency of 15 Hz [78]. We used an epoch of 1000 ms that included a pre-stimulus baseline of 200 ms and a post-stimulus signal of 800 ms. Finally, we obtained each recorded ERP over several averaging epochs ranging from 30 to 36 for each painful condition. Then, for each self-pain and empathy pain condition treatment, we calculated the ground mean ERP waveform and the time interval around the peak of each ERP component of interest, which served to detect the maximum peak amplitude. In particular, for each treatment, the time interval for the N1 was 80–140 ms, 180–240 ms for the P2, and 280–380 ms for the P3. We then identified the interval latency to use for peak area exporting, observing the maximum amplitude for each ERP component of interest on the grand average both for the pain or empathy pain condition.

It is essential to say that the P3 component immediately followed the P2 in this experiment. We believe that the P2 overlapped with the P3a, reflecting cognitive appraisal or attention to a painful stimulus [48]. Thus, it was not possible to study the N2 component during the pain or empathy pain experience. Additionally, although we were interested in exploring the P4 wave, we observed that this component was minimal and not reliably present in the ERP waveforms in the conditions of our interest. As a result, we identified only the N1, P2, and P3 waves on each ERP response. We then calculated the peak amplitude for each ERP wave using the peak export tool implemented in Brain Vision 2.1.0. statistical analysis.

We used a median split of pain difference scores to form two groups, one of the high pain reducers and the other of low pain reducers. We considered each participant as belonging to the high pain reducers or low pain reducers when their perceived pain scores were, respectively, above or below the median of this measure (*N* = 63, M = 0.59, SE = 0.127, Md = 0.41, range = 6.38). The same method was used for empathic pain (*N* = 63, M = 0.005, SE = 1.05, Md = 0.04, range = 6.51), perceived unpleasantness (*N* = 63, M = 0.40, SE = 0.13, Md = 0.40, range = 5.42), and empathy unpleasantness (*N* = 63, M = 0.21, SE = 0.084, Md = 0.12, range = 3.34) scores. The scores of the participants that fell on the median value, if any, were excluded. These clusters served to highlight how individual differences in the placebo analysis reflected on ERP wave variations. We conducted separate statistical analyses using the SAS 9.4 system. To evaluate the modulation of perceived pain and empathy for pain induced by the placebo analgesic effect on ERP waves of interest, we calculated the control minus placebo difference scores of the N1, P2, and P3 amplitudes using a separate repeated-measures analysis of covariance (ANCOVA) with the following design: 2 pain reduction levels (high, low) × 3 quadrants (left, midline, right) × 3 location (frontal, central, posterior). We performed similar analyses on the perceived pain (control minus placebo) and perceived unpleasantness difference scores and the empathy pain and empathy unpleasantness difference scores. We entered Gender and state anxiety changes as covariates. The first factor was between subjects in these analyses, while the second and third were within-subject factors. This model evaluated the placebo analgesic effect and the scalp location of these effects as within-group factors.

**Figure 2:**
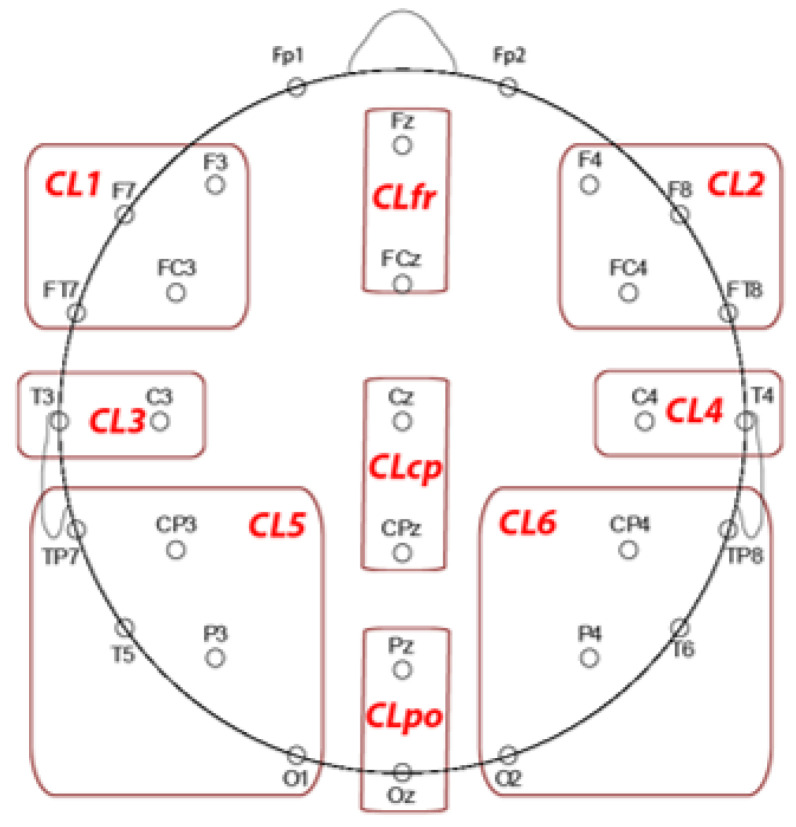
EEG sites grouped in the clusters used to perform the statistical analysis on N1, P2, and P3 components.

We reduced ERP data dimensionality for each ERP wave of interest into nine clusters (CL; see Figure 2). We labelled clusters as CL1 (left frontal), CL2 (right frontal), CL3 (left centrotemporal, CL4 (right centrotemporal), CL5 (left posterior), CL6 (right posterior), midline frontal (CLfr), midline centroparietal (CLcp) and parieto-occipital (CLpo). We calculated control minus placebo difference scores for each region and ERP peak measure of interest.

The alpha level of significance was set at 0.05, unless otherwise specified, and Huynh-Feldt adjustments [79] were applied not to compromise the assumption of sphericity. We performed two-tailed *t*-tests and post-hoc contrast analyses (α = 0.05). We assessed Pearson’s correlations to evaluate the relationships between personality traits of interest and pain changes in both self-and other-pain conditions. We applied the false discovery rate (FDR) correction method to avoid false positives. We performed a conditional process analysis [80] to test the link between ERP wave changes and placebo pain changes by entering the personality trait of interest as a simple moderator or mediator of this link. We included Gender and state anxiety changes as covariates.

Finally, the direction of the main or interaction effects involving the personality traits, electrophysiological measures, and state variables of interest was grouped for the significant factor and displayed as graphic illustrations.

## 3. Results

### 3.1. Behavioral Results

Table 1 reports Pearson correlation coefficients among personality traits and Control minus Placebo changes of perceived pain and unpleasantness in the phasic pain and empathy pain conditions.

The correlation of the FFFS score with perceived pain change and state anxiety with perceived unpleasantness change were the only that reached the significance level after FDR correction (Table 1).

**Figure 3:**
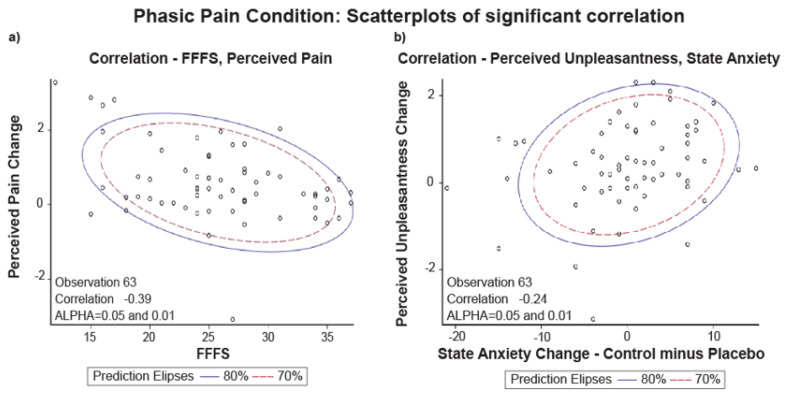
Phasic Pain Condition: (**a**) scatterplot of Pearson correlation between FFFS and Perceived Pain Change (Control minus Placebo, *N* = 63); (**b**) scatterplot of Pearson correlation between perceived Unpleasantness and State Anxiety Change (Control minus Placebo, *N* = 63).

Figure 3a shows the scatterplot of the distribution for FFFS scores with perceived pain change. Figure 3b shows the scatterplot of the distribution for state anxiety with a perceived unpleasantness change.

The scatterplots of Figure 3 show that the two variables’ data distribution is within the two ellipses (70% and 80% of the data). The displayed prediction ellipses in the figures are centered at the two variables’ means (alpha = 0.05 and 0.01). Thus, we can say that the variable FFFS scores with a perceived pain change and state anxiety with perceived unpleasantness change are normally distributed and correlated.

**Figure 4:**
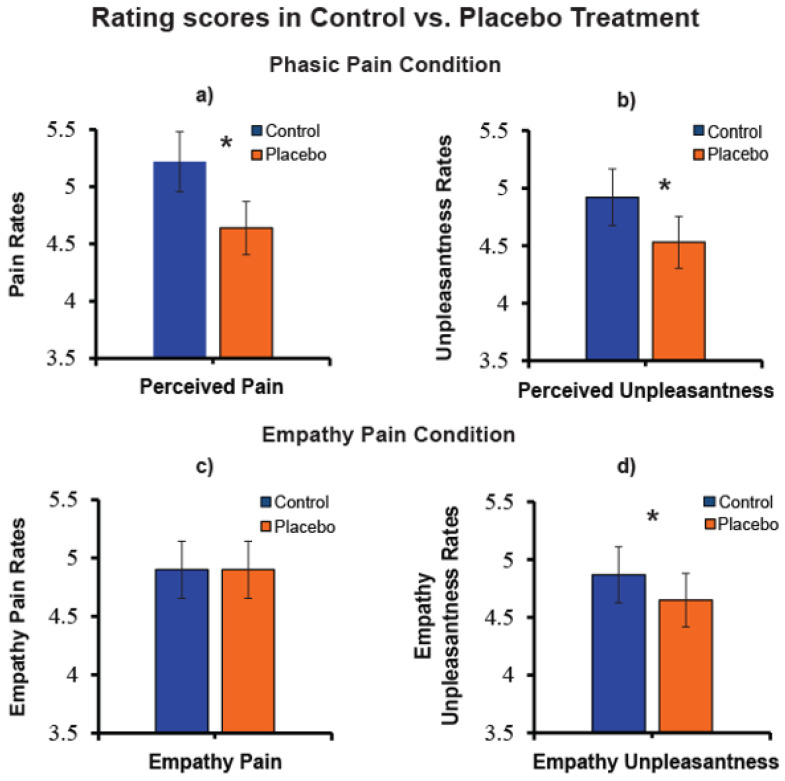
(**a**) Pain rating mean values of perceived pain in control and placebo treatment (*N* = 63); (**b**) Unpleasantness values of perceived unpleasantness mean values in control and placebo treatment (*N* = 63); (**c**) Empathy for pain mean ratings in control and placebo treatment (*N* = 63); (**d**) Empathy for unpleasantness mean ratings in control and placebo treatment.“*” Significant planned comparisons, *p* < 0.05.

To test the effect of pain reduction to placebo treatment, we performed separate *t*-tests for pairwise comparisons on rating pain and unpleasantness in the self-pain and empathy pain conditions. These analyses highlighted a significant perceived pain reduction (*N* = 63, t (62) = 4.59, *p* < 0.0001; Figure 4a), as well a perceived unpleasantness reduction (*N* = 63, t (62) = 3.17, *p* = 0.002; Figure 4b) during the treatment of placebo analgesia in the phasic pain condition. In the empathy pain condition, we failed to find a significant reduction of empathy for pain (*N* = 63, t (62) = −0.04, *p* > 0.0500; Figure 4c) while empathy for unpleasantness was significantly reduced (*N* = 63, t (62) = 2.53, *p* = 0.010; Figure 4d).

Simple *t*-tests performed separately in the high, and low perceived pain reducer’s group confirmed that the placebo effect in the high pain reducers group induced a phasic-pain reduction (t (32) = 8.83, *p* < 0.0001). In contrast, for the empathy pain, this difference was not significant (t (32) = 1.53, *p* > 0.130). In the low reducers’ group the placebo effect did not induce significant perceived pain changes (t (31) = −1.13, *p* > 0.050), or empathy for pain changes (t (31) = −1.35, *p* > 0.180).

In addition, a simple *t*-test, conducted separately for the high and low perceived unpleasantness reducers’ group, confirmed the expected placebo analgesic effect in the high unpleasantness reducers’ group for perceived unpleasantness (t (31) = 11.62, *p* < 0.0001), and empathy unpleasantness (t (31) = 3.95, *p* = 0.0004). In the low perceived unpleasantness reducers’ group, the placebo treatment induced an overestimation of perceived self-unpleasantness (t (32) = −2.40, *p* = 0.020), but not for empathy unpleasantness (t (32) = −0.44, *p* > 0.660).

These results indicated that, in the high pain and unpleasantness reducers’ group, there was a significant perceived pain and unpleasantness reduction induced by the placebo treatment. In contrast, the other pain reduction was not significant in the high-pain reducers’ group.

### 3.2. ERP Results

Table 2 summaries all the results of ANCOVA calculated on N1, P2, and P3 ERP responses for pain and unpleasantness perceived in the phasic pain and empathy pain conditions, during control and placebo treatments. Finally, gender and state anxiety were entered as covariates.

#### 3.2.1. N1 Amplitude

**Figure 5:**
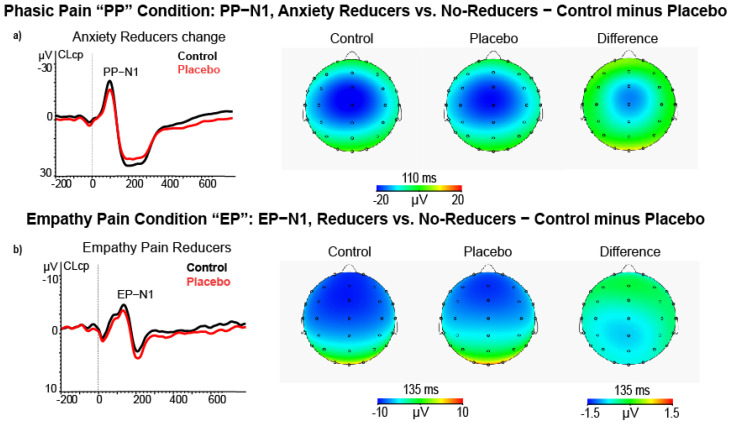
(**a**) ERP, Scalp maps and difference (control minus placebo) maps of PP−N1 amplitude for control and placebo treatments in high state anxiety reducers (*N* = 26); (**b**) ERP, Scalp maps, and difference maps of EP−N1 amplitude in other pain reducers (*N* = 31) during the empathy pain condition.

In the phasic pain condition, the ANCOVA on N1 amplitude differences scores, using high and low Pain Reduction Levels as a between-subjects factor, did not significantly affect this factor. However, this analysis yielded a significant interaction of Quadrant × Location × Anxiety change (F (4232) = 4.12, *p* = 0.007, ${ƞ}_{p}^{2}$ = 0.066). This effect showed that during the phasic pain condition, placebo treatment induced a relative reduction of N1 amplitude in anxiety reducers at midline central-parietal cluster (CLcp, t = 2.30, *p* = 0.025) with a relative decrease in state anxiety (r = −0.277, *p* < 0.050; see Figure 5a). We failed to detect significant effects involving perceived unpleasantness levels obtained for N1 amplitude change scores.

Additionally, in the empathy pain condition, the ANCOVA on N1 amplitude difference scores comparing high and low Empathy in Pain Reduction scorers yielded a main effect of empathy for pain condition (F (1, 58) = 4.52, *p* = 0.038, ${ƞ}_{p}^{2}$ = 0.072). This effect indicated that, during placebo treatment, high empathy pain reducers had a smaller N1 peak amplitude than low ones (M = −0.80, SE = 0.64 vs. M = 0.88, SE = 0.54, for control minus placebo differences scores of high-Pain Reducers vs. low-Pain Reducers, see Figure 5b).

The ANCOVA for the high and low unpleasantness level scorers did not yield any significant effect.

#### 3.2.2. P2 Amplitude

**Figure 6:**
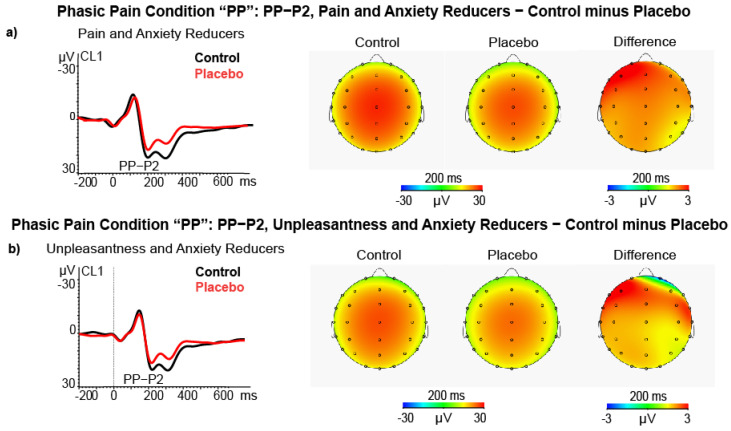
(**a**) ERP, Scalp maps and difference maps of PP−P2 amplitude in high pain and state anxiety reducers (*N* = 15); (**b**) ERP, Scalp maps and difference maps of PP−P2 amplitude in high pain and state unpleasantness reducers (*N* = 15).

In the phasic pain condition, the ANCOVA on P2 amplitude difference scores, using Pain Reduction Level as a between-subjects factor disclosed the Quadrant × State Anxiety change × Pain Reduction Level (F (2116) = 6.57, *p* = 0.005, ${ƞ}_{p}^{2}$ = 0.100; see Figure 6a). This effect showed that participants classified as high Pain Reducers and Anxiety Reducers also had a significant P2 amplitude reduction at the right central-temporal cluster (CL4, t = 2.20, *p* < 0.050) and at the left frontal cluster (CL1, t = 2.13, *p* < 0.050) during placebo treatment. The ANCOVA using Unpleasantness Level as a between-subjects factor yielded a significant interaction of Quadrant × state Anxiety change × Unpleasantness Level (F (2116) = 7.69, *p* = 0.002, ${ƞ}_{p}^{2}$ = 0.117; see Figure 4b). This effect showed that participants classified as high Unpleasantness Reducers and Anxiety Reducers also had a significantly P2 amplitude reduction at the left frontal cluster (CL1, t = 1.98, *p* < 0.050), during placebo treatment.

ANCOVAs performed for Empathy Pain and Unpleasantness Reduction Level did not yield any significant effect.

#### 3.2.3. P3 Amplitude

**Figure 7:**
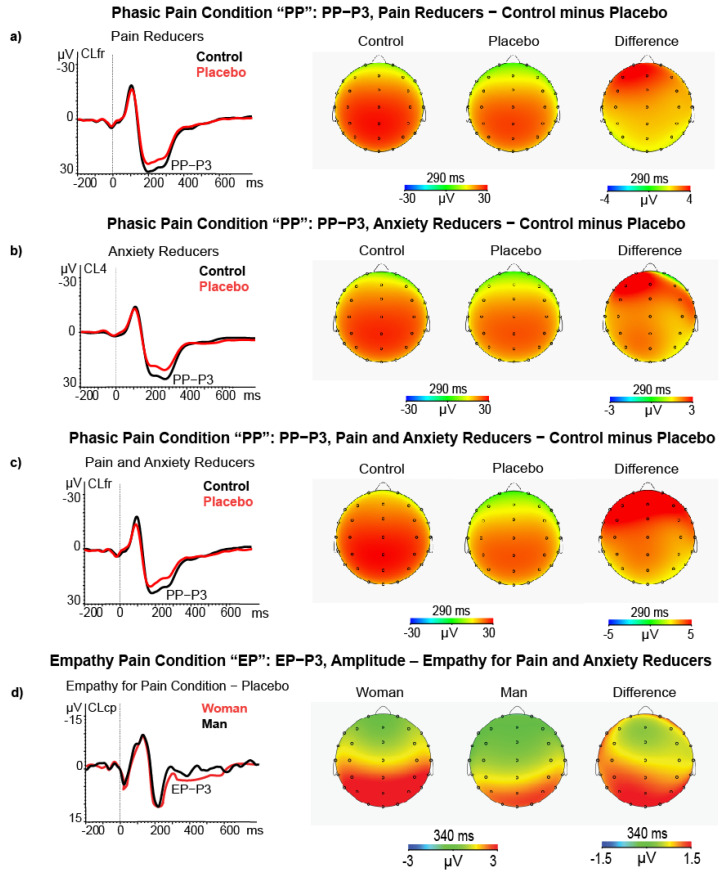
(**a**) ERP, Scalp maps and difference (control minus placebo) maps of PP−P3 amplitude in high pain reducers (*N* = 32); (**b**) ERP, Scalp maps, and difference maps of PP−P3 amplitude for control and placebo treatments in high state anxiety reducers (*N* = 26); (**c**) ERP, Scalp maps and difference maps of PP−P3 amplitude in high pain and state anxiety reducers (*N* = 15); (**d**) ERP, Scalp maps and difference maps of EP−P3 amplitude in women (*N* = 32) vs. men (*N* = 31) during the empathy pain condition.

In the phasic pain condition, the ANCOVA on P3 amplitude differences yielded a main effect for Pain Reduction Level (F (1, 58) = 4.75, *p* = 0.033, ${ƞ}_{p}^{2}$ = 0.076) and for the covariate State-Anxiety change (F (1, 58) = 4.53, *p* = 0.038, ${ƞ}_{p}^{2}$ = 0.072). The first effect indicated high Pain Reducers, as compared with Low Pain Reducers, and had a significant P3 amplitude reduction during the placebo treatment (M = 2.62, SE = 1.04 vs. M = −0.52, SE = 1.06, respectively; see Figure 7a). The second effect showed a significant positive association between State Anxiety change and P3 amplitude reduction (r = 0.303, *p* < 0.050, after FDR correction). This effect is displayed in Figure 7b. In addition, the interaction of Quadrant with State Anxiety change was also significant (F (2116) = 3.84, *p* = 0.024, ${ƞ}_{p}^{2}$ = 0.062), indicating that the highest relation was between State Anxiety change and P3 amplitude reduction was at the CL4 (t = −2.43, *p* = 0.018; r = 0.369, *p* < 0.01, FDR correction).

Finally, the third-order interaction of Location × Pain Reduction Level × State Anxiety change was significant (F (2116) = 7.97, *p* = 0.003, ${ƞ}_{p}^{2}$ = 0.120). This effect indicated that the P3 amplitude reduction was significant for high Pain Reducers who also had a State Anxiety reduction during placebo treatment. The P3 amplitude reduction was more pronounced at left-frontal (CL1, t = 3.18, *p* = 0.006), left-centrotemporal (CL3, t = 2.73, *p* = 0.016), frontocentral midline (CLfr, t = 2.73, *p* = 0.016), and mostly at the right centrotemporal cluster (CL4, t = 3.31, *p* = 0.0051; see Figure 7c). The ANCOVA used the Unpleasantness Reduction Level as a between-subjects factor but did not yield significant effects involving individual differences on Unpleasantness Reduction.

In the empathy pain condition, the ANCOVA for high and low Empathy Pain Reduction groups on the P3 amplitude difference scores yielded a main effect for the covariate Gender (F (1, 58) = 4.90, *p* = 0.031, ${ƞ}_{p}^{2}$ = 0.078) and for Quadrant (F (2116) = 24.1, *p* < 0.0001, ${ƞ}_{p}^{2}$ = 0.296) and for Quadrant × Location (F (4232) = 4.70, *p* = 0.001, ${ƞ}_{p}^{2}$ = 0.078). The Gender effect yielded higher P3 amplitude during placebo than control treatment in women compared to men (*N* = 32, M = −5.82, SE = 0.86 vs. *N* = 31, M = 2.82, SE = 0.98, respectively; see Figure 7d).

The Quadrant × Location significant effect (F (4232) = 4.70, *p* = 0.001, ${ƞ}_{p}^{2}$ = 0.075) showed that after the administration of placebo treatment the left frontal cluster had a relative smaller P3 amplitude enhancement than the midline central-parietal-temporal and parieto-occipital ones (*N* = 63, M = −1.83, SE = 0.46 vs. M = −4.42, SE = 0.62 and M = 4.82, SE = 0.52, respectively) as well as for the right frontal and right centrotemporal cluster versus midline centroparietal and parieto-occipital clusters (*N* = 63, M = −1.89, SE = 0.42, and M = −1.86, SE = 0.44 versus M = −4.42, SE = 0.62 and M = 4.82, SE = 0.52). All comparisons (*t*-test) were significant (*p* < 0.001). In addition, the Quadrant × Location × Gender was also significant (F (4232) = 3.63, *p* = 0.011, ${ƞ}_{p}^{2}$ = 0.059) and disclosed that, during placebo treatment, the women had a significantly higher P3 amplitude (i.e., higher negative difference scores) than men at left-frontal cluster CL1 (t = −2.40, *p* = 0.019), left centroparietal cluster CL3 (t = −2.21, *p* = 0.031), midline frontal cluster CLfr (t = −2.38, *p* = 0.020), midline centroparietal CLcp (t = −2.39, *p* = 0.020), right fronto-central cluster CL2 (t = −2.01, *p* = 0.049) and right temporal-parietal-occipital cluster CL5 (t = −2.01, *p* = 0.028).

The analysis for Empathy Unpleasantness as a between-subject factor failed to find a main or interaction effect involving the Unpleasantness Reduction factor.

#### 3.2.4. ERP Waves and Personality Influence on Placebo Pain Reduction

**Figure 8:**
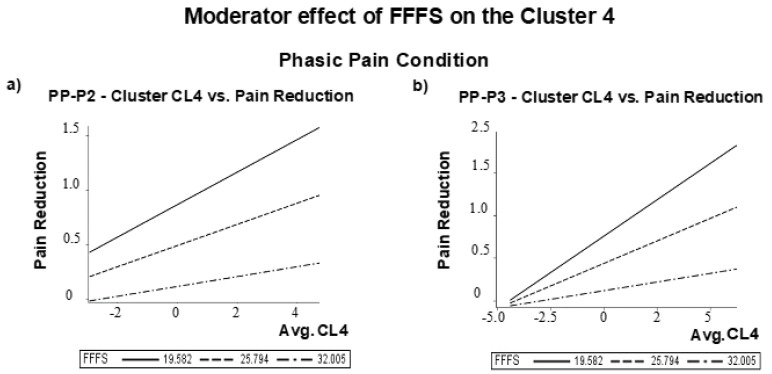
Moderator effect of FFFS on the Cluster 4 (CL4) for the Phasic Pain condition: difference vs. Pain reduction for (**a**) PP-P2 amplitude; (**b**) PP-P3 amplitude difference scores.

As previously reported in Table 1, among Pearson correlation coefficients between RST-PQ personality traits and pain and unpleasantness changes after the placebo treatment, the FFFS was the only trait significantly correlated with perceived pain reduction in phasic pain condition. In addition, we found that P2 amplitude difference scores at right centrotemporal cluster CL4 (r = 0.38, *p* = 0.048) and P3 amplitude difference scores at right centrotemporal cluster CL4 (r = 0.50, *p* < 0.001) were the only two significantly correlated with perceived pain reduction after FDR correction. In the phasic pain condition, the correlation of perceived pain reduction with P3 amplitude changes at right temporal-parietal-occipital cluster CL6 was also significant (r = 0.40, *p* = 0.007) as well as with P3 changes at frontal and central-parietal clusters (CLfr and CLcp) were both significant, although at a lower level of significance (r = 0.38, *p* = 0.011, and r = 0.38, *p* = 0.010, respectively after FDR corrections). On these bases, we first tested how P2 amplitude changes at CL4 to placebo treatment influenced placebo analgesia and how the FFFS moderates this effect. This analysis was performed using the conditional process analysis (PROCESS, model 1), as suggested by Hayes [80], with Gender and State-Anxiety changes entered as covariates. We supplemented this analysis with the 95% percentile Class Interval (CI) on 5.000 bootstrap samples. The model was highly significant (R^2^ = 0.353, F (5, 57) = 6.22, *p* = 0.0001). Both P2 amplitude changes and FFFS scores significantly influenced self-pain reduction, although the conditional interaction effect of FFFS with P2 amplitude changes did not reach significance (see Table 3). However, using the quantile = 1 command line in the PROCESS command, we obtained that the conditional moderator effect of FFFS linking P2 amplitude changes to pain reduction was significant among low to moderate FFFS values. The moderator values, defining Johnson-Neyman significance regions, ranged from FFFS = 12.0 to 28.25 (i.e., from 25.4% to 74.6% of the FFFS value of 29.37; t values ranged from 2.5 to 3.9; *p* values ranged from 0.02 to 0.0003). Figure 8a shows this conditional moderator effect.

We obtained significant correlations of pain reduction with P3 difference scores at CL4, CL6, CLfr, and CLcp clusters. We then calculated the correlation matrix among these four potential electrocortical predictors of perceived pain reduction. We found that all the intercorrelations ranged between 0.87 and 0.94 and indicated multicollinearity. We then assessed collinearity diagnostics as supplied by Proc Reg (SAS-9.4). In this multiple regression procedure, we used as predictors the P3 difference values at CL4, CL6, CLfr, and CLcp clusters and perceived pain reduction as the criterion. We obtained that only P3 differences at cluster CL4 had a Variance Inflation Factor (VIF) of 0.16 and a Tolerance (TOL) of 6.40 (i.e., above the conventional cut off of 0.10 and below the cut off value of 10, respectively), whereas for the measures at cluster CL6, CLfr and CLcp were out of the abovementioned conventional limits. For the sake of completeness, we also reviewed the eigenvalue and condition index association. We observed that only the first factor (i.e., only P3 differences at CL4) had an eigenvalue closer to 1, whereas the other measures were zero. We also noted that the P3 scores at CL6, CLfr, and CLcp had a proportion of covariation ranging from 0.63 to 0.83. Thus, we selected the P3 difference score at cluster CL4 for a further conditional process analysis [80].

Based on these results, we tested how the influence of placebo-induced P3 changes at cluster CL4 on pain reduction is conditionally moderated by the FFFS trait [80] using gender and state anxiety changes as covariates. The model was tested as highly significant (R-square = 0.446, F (5, 57) = 9.19, *p* = 0.00001). The effects of P3 changes, FFFS scores, and their interaction were all significant (see Table 3. This analysis yielded that the conditional moderator effect of FFFS in the relation linking P3 amplitude changes to pain reduction was significant among low to moderate FFFS values. Moderator values defining Johnson-Neyman significance regions ranged from FFFS = 12.0 to 30.75 (i.e., from 19.04% to 80.95% of the FFFS value of 31.14; t values ranged from 2.5 to 5.2; *p* values ranged from 0.03 to 0.00001). The graphic of Figure 8b depicts this conditional effect using values of the FFFS sample mean and with those of 1 SD below and above the FFFS mean.

## 4. Discussion

The results of the present study confirmed previously reported findings by Rütgen and colleagues [38] of a placebo-induced reduction of self-pain and self-unpleasantness. Still, we failed to find a drop of other pain. In particular, the empathy pain reduction was not significant in the group of high-pain reducers. Thus, we failed to confirm a reduction in empathy pain, as previously reported by Nir and colleagues [81]. In terms of placebo-induced empathy effects, we found a small but significant, other-unpleasantness reduction.

In their experiments, Rütgen and colleagues [38] and Mischkowski and colleagues [36] assessed the effect of placebo analgesia treatment on the public domain of perceived pain and empathy for pain using only the pain rating. They lacked the measurement of the emotional component of pain. In this regard, it is essential to state that studying empathy pain and ignoring empathy unpleasantness can represent a limit. Fields [6] highlighted the necessity to differentiate primary unpleasantness from both algosity (a quality that allows the identification of some somatic sensations as pain) and secondary unpleasantness (a higher-level process that is determined mainly by memories and contextual features). We believe that empathy for pain would be better conceptualized using the differentiation of pain’s sensory and emotional components. Research has also shown that observing others suffering from physical pain evokes empathic reactions, including emotions and feelings that can lead to prosocial behavior and might be regarded as the social value of pain [82]. Moreover, considering that empathy for unpleasantness is related to the activity of brain regions that are different from those involved in empathy for pain [83], we urge researchers to further study placebo-induced modulation on the empathy for pain and unpleasantness responses.

From a neurophysiological point of view, our findings suggest that placebo analgesia treatment significantly reduced the amplitude of the N1, P2 and P3 ERP components elicited by painful stimulation.

In terms of the negative ERP components, research has highlighted that the N1 (125–155 ms) and N2 (230–260 ms) components of the ERP evoked by phasic pain stimulation reach the maximal amplitude in the frontocentral brain regions [57,59,84,85]. In this regard, our results highlight that the placebo induced a perceived pain and state anxiety modulation associated with N1 amplitude reduction in the midline central brain region. Our results show an empathic pain reduction induced by the placebo in the empathy pain reducer group for the empathy pain condition. Our N1 wave, evoked by the empathy pain stimulus, varied in latency between 80 and 140 ms in this condition. Considering that the latency was inferior to 200 ms, we classified this negative deflection as an N1. This finding aligns with Valeriani and colleagues’ [86] observations that images in which others received phasic pain stimulation modulate the N1 component that originated in the secondary somatosensory cortex and related to the discrimination of sensory and affective pain components [60,87]. In particular, this research disclosed that during an empathy pain task, the N1 response on the frontocentral brain areas of participants could reflect automatic activation of the affective arousal or emotional sharing component of inferred pain [85,88,89].

In contrast, the N2 response could indicate more late controlled processes of others’ pain evaluation during the empathy pain condition [59,89]. Moreover, the N2 component originates in the middle anterior cingulate cortex (mACC) [39]. This brain region is involved in attentional shift and orienting processes [90], and in both first-hand experience of pain [91] and empathy for pain [37], and placebo analgesia [92].

In line with the research mentioned above, we can already assume that from the first stages of stimulus processing (in addition to the processes of orientating and discrimination of the stimulus), our N1 component reflects a cognitive function related to the estimation of empathy pain identification.

We also observed a significant effect of placebo analgesia with state anxiety interaction on ERP amplitude reduction. Some research had suggested that the state anxiety can increase the amplitude of the N1 component [93], influencing spinal cord neuron processing before the painful stimulus was delivered [94]. In this vein, enhanced state anxiety could reflect enhanced anticipatory arousal adaptive process [95] to afford the oncoming painful stimulation [96].

Our findings indicate that placebo treatment during the perceived pain condition induced a relative reduction of the N1 amplitude at the midline central-parietal cluster (near the parietal operculum) [48] in parallel with a relative decrease of state anxiety. Thus, placebo analgesia can reduce pain-related stress [73,97] by decreasing pain perception during the early phase of nociception. The inhibitory modulation of the N1 component is related to both the subjective measures of sensory and the emotional qualities of pain and the feelings derived from observing stimuli delivered to others. Thus, it seems to mediate the linkage between first-hand pain sensitivity and empathic behavioral responses [98]. The N1 component is a marker of the automatic activation of affective arousal or emotional sharing [99]. Thus, the placebo effect can reduce the attentional and discriminative processes indexed by the N1 wave [46], associated with the early phase of sensory pain and the empathic sensory component of pain.

Regarding positive late ERP components, similar to Rütgen and colleagues [38], we observed a P2 peak of maximum amplitude at the central brain sites near the somatosensory cortex. However, we note that the P2 wave detected by these authors was in a latency range of 200–300 ms, which is typical for the early P3. In the present study, P2 was observed only in the perceived pain condition, followed by another positive deflection classifiable in latency as a P3. However, from a conceptual point of view, in terms of peak latency, ERP shape, and location, it may be that our two positive peaks can also be classified as P3a and P3b sub-components. The label of P3a seems appropriate, considering that the P3a reaches its maximum amplitude on frontal–central brain regions, while the P3b comes to its maximum amplitude on temporal-parietal brain regions [100]. In this regard, in terms of self-pain, we found reductions of the P2 amplitudes of the ERPs induced by placebo analgesic effects on the left frontal hemisphere, in high pain and unpleasantness reducers and state anxiety reducer groups. The P2 wave is associated with the intensity of subjective pain perception [101,102] and reflects the salience of relevant stimuli [103,104]. Our P2 electrophysiological findings confirm a sensory modulation of the nociceptive input [45] induced by placebo analgesia. However, it is difficult to determine the causal relationship between perceived pain or unpleasantness reduction and state anxiety reduction. Therefore, similar to the N1 modulation described above, placebo analgesia treatment could have reduced the arousing processes related to state anxiety and indirectly involving the prefrontal cortex [17] to act consequently on pain reduction in the participants that accepted the treatment [105].

Regarding the P3 component, during the perceived pain condition, our findings disclosed a placebo-induced amplitude reduction of this ERP component in high pain reducers, compared with low ones, and in the group of participants who were both high pain and anxiety reducers. In this last group, we observed a placebo-induced reduced P3 wave in the brain overall.

Moreover, Decety [85] argued a significant and diffused higher P3 amplitude after placebo treatment in women than men regarding the empathy pain condition. This result is consistent with previous findings [106] and highlights a relatively higher cognitive processing involvement in women participants.

In line with the studies above, we can conclude that placebo-induced reductions of P2 and P3 amplitudes reflect a decrease in sustained attention [100] and changes in the cognitive evaluation of motivationally relevant stimuli [107]. In this sense, phasic pain might contribute to understanding the late controlled social interaction and emotional regulation [84,108]. In summary, these findings support a significant reduction of P2 and P3 components related to perceived pain and unpleasantness, rather than an empathy pain or unpleasantness reduction induced by placebo.

The lack of results referring to the relationship between the empathic response and electrophysiological modulation induced by placebo can be due to different factors, such as the experimental context and intervention variables. Besides, research has suggested that a threatening context could suppress the empathic response to other-pain or -unpleasantness. For example, Meng and colleagues [57] disclosed that the P3 amplitudes were significantly decreased in response to the empathy pain condition when presented with the pain cue. In this regard, although we delivered the phasic pain stimulation to participants and confederate in random order in the current study, we can assume that first-hand pain was more salient than empathy pain perceived by the other participant for each participant. Furthermore, we think that the incremental role of state anxiety, generated by the painful oncoming stimulation, could have generated an aversive response opposite to that of the empathic expected response. From an evolutionary perspective, state anxiety could down-regulate the sensory and cognitive processing elicited by perceiving others’ pain [109].

In contrast, the processing of painful stimuli associated with a potential threat first activates the threat detection system [110]. In this case, self-survival is more salient than any other survival [98]. Our empathy findings indicate that the context and psychological state impair our ability to empathize with others’ pain [111].

Concerning the relationship between the approach/avoidance personality traits [22] and placebo-induced modulation during the phasic pain and empathy pain conditions, we found that the FFFS is the only motivational trait associated with perceived pain reduction induced by placebo analgesia.

We used conditional process analysis to find that the relative P2 and P3 amplitude reductions at the right central-temporal scalp regions, induced by placebo treatment, influenced perceived pain decline by moderating FFFS scores. These influences reached the significance level for low to moderate FFFS scores (Figure 7a,b). These cortical regions, including the somatosensory cortex and MCC [112], are part of the µ-opioid system, which is associated with the modulation of perceived pain and placebo analgesia [113]. In this vein, it is significant that placebo analgesia can reduce both the P2 and P3 components, respectively related to the early focal attention to pain stimulus and the subsequent memory comparison that is served by a circuit connecting the frontal and temporal-parietal brain regions [100]. In particular, the dorsal-lateral-frontal cortex, inferior frontal cortex, and anterior insular cortex are suggested to be part of the same system [114]. These brain areas with the anterior insular cortex are part of multiple interactive processes, including pain localization and quantification [115,116], the experience of pain in terms of negative and emotionally affective stimulation [117], and the observation of others in pain [50,118]. The above-reported moderation effects of FFFS align with previous statements suggesting that the placebo effect can modulate both negative affect and fear [32]. In conclusion, our findings indicate that both low FFFS scores and the relatively smaller P2 and P3 amplitudes at the right central–temporal brain regions induced by the placebo treatment can account for perceived pain reduction. Our findings align with the r-RST view that phasic pain is an aversive stimulus eliciting active avoidance behavior, necessary to restore the system to homeostasis [30].

These results have important clinical implications that can be briefly summarized. It is known that patients affected with chronic or neuropathic pain do not respond to pharmacological treatments [119]. However, recent research has suggested that individuals with chronic pain exhibit a similar placebo analgesic response (magnitude and reliability) to that exhibited by healthy controls [120]. Suppose our results are also valid for patients suffering from chronic pain with relatively low levels of active avoidance or fear personality traits. In that case, researchers can consider alternative management using a placebo analgesic treatment [121]. These results advocate that in their design for pain treatment targets, researchers should not forget to consider fear-related motivational personality traits of their patients.

Similar to any study, this experiment has some limitations. One potential limitation is the small number of the sample. Furthermore, the present sample was drawn from neurotypical right-handed university students. It would also be essential to determine whether the current observed findings would be consistent in the general population of different age ranges. Moreover, recent research highlights the relevance of studying the placebo-induced modulation of the unpleasant stimuli disclosed as the placebo effect that can reduce empathy unpleasantness [83]. Since our interest was to study phasic pain modulation induced by placebo treatment, we did not focus on the no pain stimulation classified as unpleasant. In our current experiment, we used a painful stimulation method to control uniquely for pain habituation.

Furthermore, some recent ERP research has outlined that (a) the P3 is enhanced with increased predictability and probability, (b) this ERP component is positively correlated with empathy trait scores, and (c) the more expected pain of others can trigger more robust empathic responses reflected in the increased amplitudes of P3 [122,123]. On this basis, we expect that future research should further evaluate this relationship by exploring how the increased expectation of others’ pain can enhance subsequent prosocial behavior. Although our findings encourage understanding the electrocortical mechanisms of placebo-induced reductions on pain and empathy pain responses, they should be treated with caution. Experimental manipulations or state variable modulations could impair this relationship and influence individual emotional states or cognitive processes [54]. We hope that future research will aim to gain a further focus on these results and assumptions.

## Figures and Tables

**Table 1:** Pearson correlations and descriptive statistics for behavioral and personality measures in 63 participants.

	*Pearson Correlation Coefficients, N = 63 > |r| under H0: Rho = 0*							
Average Control Minus Placebo	BAS TOT ^a^	BAS GDP ^b^	BAS RI ^c^	BAS RR ^d^	BAS Imp ^e^	BIS ^f^	FFFS ^g^	STAI-Y1 ^h^ Change
Perceived Pain Change	−0.05	−0.05	−0.11	0.06	−0.06	−0.15	−**0.39** •	0.16
Perceived Unpleasantness Change	−0.02	−0.05	−0.12	0.01	0.08	−0.04	−0.14	**0.24** •
Empathy for Pain Change	0.06	0.08	0.08	0.03	0.01	−0.01	−0.13	−0.09
Empathy for Unpleasantness Change	0.09	0.02	−0.09	0.16	0.13	0.08	−0.07	−0.16

**•***p* < 0.05, FDR correction. ^a^ Behavioral Approach System-total; ^b^ Behavioral Approach System-Goal Drive Persistence; ^c^ Behavioral Approach System-Reward Interest; ^d^ Behavioral Approach System-Reward Reactivity; ^e^ Behavioral Approach System-Impulsivity; ^f^ Behavioral Inhibition System; ^g^ Fight-Flight-Freeze System; ^h^ State Anxiety Inventory–Scale Y1.

**Table 2:** Significant effects detected by ANCOVA (Gender and State Anxiety were entered as covariates).

<Amplitude Change (Pain Minus Placebo)	N1				P2		P3			
Condition	*Phasic* *Pain*		*Empathy Pain*		*Phasic* *Pain*		*Phasic* *Pain*		*Empathy* *Pain*	
ANCOVA	F	*p*	F	*p*	F	*p*	F	*p*	F	*p*
Pain Reduction	-	-	-	-	-	-	4.75	0.033	-	-
Empathy Pain Reduction	-	-	4.52	0.038	-	-	-	-	-	-
Empathy Pain Reduction × Quadrant	-	-	-	-	-	-	-	-	24.1	<0.0001
Empathy Pain Reduction × Quadrant × Location	-	-	-	-	-	-	-	-	4.70	0.001
Main effect for State Anxiety	-	-	-	-	-	-	4.53	0.038	-	-
Quadrant × Location × State Anxiety	4.12	0.007	-	-	-	-	-	-	-	-
Quadrant × State Anxiety × Pain Reduction Level	-	-	-	-	6.57	0.005	7.97	0.003	-	-
Quadrant × State Anxiety × Unpleasantness Level	-	-	-	-	7.69	0.002	-	-	-	-
Main effect for Gender	-	-	-	-	-	-	-	-	4.90	0.031

“-“ non-significant values.

**Table 3:** Multiple regression of cluster 4, CL4 (C4, T4 EEG sites) on FFFS RST-PQ trait.

	*Simple Moderation Analysis Model*			
	(a) PP-P2 component			
CI = 95%	B	se	*t*	*p*
Intercept	2.14	0.630	3.39	0.001
FFFS ^g^	**−0.06**	**0.021**	**−2.83**	**0.006**
Avg. CL4	0.31	0.128	2.44	0.018
CL4 × FFFS	−0.01	0.005	−1.71	0.092
GENDER	−0.20	0.251	−0.79	0.435
STAI-Y1 C-P ^h^	0.01	0.016	0.911	0.366
	**(b) PP-P3 component**			
Intercept	1.83	0.570	3.21	0.002
FFFS ^g^	**−0.05**	**0.019**	**−2.70**	**0.009**
Avg. CL4	0.38	0.108	3.48	0.001
CL4 × FFFS	−0.01	0.004	−2.70	0.009
GENDER	−0.14	0.230	−0.60	0.552
STAI-Y1 C-P ^h^	−0.01	0.015	−0.60	0.550

^g^ Fight-Flight-Freeze System; ^h^ State Anxiety Inventory–Scale Y1 (Control minus Placebo).

## Data Availability

Data of this study were collected at “La Sapienza” University of Rome (Italy).
